# Targeting of Protein Persulfidation in Aging and Age-Related Disorders

**DOI:** 10.3390/pharmaceutics18101266

**Published:** 2026-10-07

**Authors:** Martín Hugo, Javier Garcia-Pardo

**Affiliations:** 1 Departament de Bioquímica i Biologia Molecular, Universitat Autònoma de Barcelona, Bellaterra, 08193 Barcelona, Spain; 2 Institut de Biotecnologia i Biomedicina, Universitat Autònoma de Barcelona, Bellaterra, 08193 Barcelona, Spain

**Keywords:** aging, biomolecular condensates, ergothioneine, healthspan, hydrogen sulfide, liquid–liquid phase separation, oxidative stress, neurodegeneration, persulfidation, protein aggregation, cysteine hyperoxidation, cysteine sulfonylation-mediated aggregation

## Abstract

Aging is associated with a progressive loss of proteostasis and increased susceptibility to neurodegenerative and metabolic disorders. Among the molecular mechanisms implicated in these processes, protein persulfidation, a hydrogen sulfide (H_2_S)-mediated cysteine modification, has emerged as a critical regulator of protein function and redox homeostasis. Recent studies demonstrate that aging is accompanied by a decline in persulfidation and a concomitant increase in irreversible cysteine hyperoxidation, leading to protein dysfunction and aggregation. Beyond its established role in protecting proteins from oxidative damage, persulfidation has recently been shown to regulate biomolecular condensation and liquid–liquid phase separation, suppressing the formation of pathological protein assemblies linked to aging and neurodegenerative diseases. Moreover, pharmacological approaches that enhance persulfidation, including stimulation of endogenous H_2_S production and treatment with ergothioneine, improve metabolic resilience, increase NAD^+^ levels, and promote healthspan in experimental models. In this review, we discuss emerging evidence linking loss of persulfidation to protein aggregation, biomolecular condensates, and age-related functional decline, and highlight therapeutic strategies aimed at restoring persulfidation as a promising avenue for healthy aging and disease intervention.

## 1. Introduction: Protein Persulfidation in Aging and Age-Related Disorders

Aging is characterized by a progressive decline in cellular homeostasis driven by the accumulation of molecular damage, impaired stress responses, metabolic dysfunction, and the gradual loss of proteome integrity [1,2,3]. Among the molecular alterations associated with aging, dysregulation of redox signaling has emerged as a central mechanism contributing to functional decline and the development of age-related disorders [2,4]. Rather than representing solely a consequence of excessive reactive oxygen species (ROS) production, aging is increasingly recognized as a disruption of finely regulated oxidative post-translational modifications (PTMs) that normally coordinate protein function, metabolism, and stress adaptation [5,6,7].

Among the endogenous signaling systems that regulate these redox modifications, hydrogen sulfide (H_2_S) has recently gained considerable attention as a central regulator of cysteine redox biology [7]. Initially regarded simply as a toxic gas, H_2_S is now recognized as an important regulator of cellular metabolism, inflammation, stress responses, mitochondrial function, and longevity, alongside nitric oxide (NO) and carbon monoxide (CO) [8,9]. In mammalian cells, endogenous H_2_S is primarily synthesized by cystathionine β-synthase (CBS), cystathionine γ-lyase (CSE), and the coupled cysteine aminotransferase (CAT)/3-mercaptopyruvate sulfurtransferase (3-MST, MPST) pathway, although the relative contribution of each pathway varies among tissues and physiological conditions [10,11,12,13]. While H_2_S can interact directly with metal centers, reactive oxygen and nitrogen species, and mitochondrial electron transport components, a substantial body of evidence now supports protein persulfidation (also referred to as S-persulfidation) as the principal molecular mechanism through which H_2_S exerts many of its biological functions [7,14,15]. Protein persulfidation involves the conversion of a cysteine thiol (–SH) into a persulfide (–SSH), generating a chemically distinct sulfur species with enhanced nucleophilicity and altered redox properties [16].

Unlike irreversible oxidative modifications, persulfidation is a reversible and dynamically regulated process, allowing it to function as a bona fide signaling PTM analogous to phosphorylation or S-nitrosylation [15,17,18]. Beyond regulating enzyme activity, protein–protein interactions, and intracellular signaling pathways, persulfidation plays an essential cytoprotective role by preserving cysteine residues in a redox-active yet reversible state. Oxidative modification of protein thiols typically proceeds through sulfenic acid (–SOH), which, under physiological conditions, may either be reduced back to thiols or further hyperoxidized to sulfinic (–SO_2_H) and ultimately sulfonic acids (–SO_3_H). While sulfenylation can function as a transient signaling intermediate, hyperoxidation is generally irreversible and frequently associated with protein dysfunction and enzyme inactivation [2,15,19,20]. Persulfidation provides an effective mechanism to intercept this process by transiently modifying reactive cysteines into persulfides, thereby shielding them from irreversible hyperoxidation while maintaining their regulatory capacity [7]. However, a growing body of evidence indicates that this protective mechanism progressively deteriorates during aging [21,22,23]. Specifically, this indicates that physiological aging is accompanied by a progressive decline in protein persulfidation together with the accumulation of oxidative cysteine modifications, including sulfenylation and irreversible hyperoxidation, reflecting extensive remodeling of the cysteine redox proteome [7,24,25].

**Figure 1:**
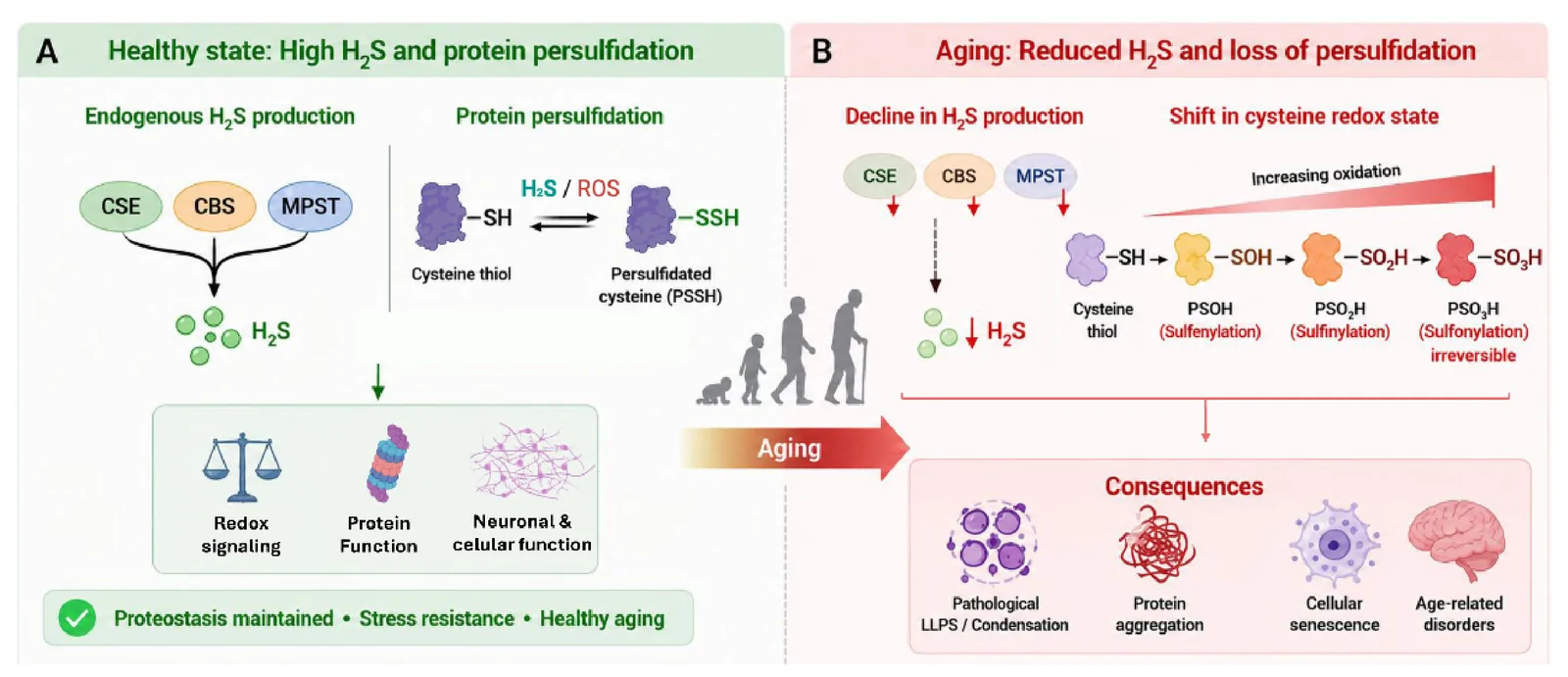
Protein persulfidation as a regulator of proteostasis during aging. (**A**) Under normal physiological conditions, endogenous hydrogen sulfide (H_2_S), produced mainly by cystathionine γ-lyase (CSE), cystathionine β-synthase (CBS), and 3-mercaptopyruvate sulfurtransferase (MPST), promotes reversible protein persulfidation (PSSH) of reactive cysteine residues. Persulfidation protects cysteines from irreversible oxidation while modulating protein function and cellular homeostasis. (**B**) During aging, endogenous H_2_S production declines, leading to reduced protein persulfidation. As PSSH levels decrease, reactive cysteine residues become increasingly susceptible to oxidation, shifting the cysteine redox state toward sulfenylation (PSOH), sulfinylation (PSO_2_H), and ultimately irreversible sulfonylation (PSO_3_H). These changes in cysteine redox homeostasis have been linked to impaired proteostasis, protein aggregation, mitochondrial dysfunction, cellular senescence, and age-associated disorders.

Only recently, quantitative chemoproteomic profiling of the aging mouse brain provided direct proteome-wide evidence for this process, revealing a widespread reduction in protein persulfidation accompanied by a concomitant increase in protein sulfenylation [21]. These alterations affected thousands of proteins involved in energy metabolism, synaptic transmission, neuronal signaling, cytoskeletal organization, and protein homeostasis (proteostasis), revealing extensive remodeling of redox-regulated cellular pathways during brain aging. Furthermore, similar redox remodeling was observed in CSE-deficient mice, in which reduced endogenous H_2_S production accelerated the loss of protein persulfidation and promoted oxidative cysteine remodeling, providing compelling evidence that impaired sulfide signaling contributes directly to age-associated proteome dysfunction (Figure 1). Moreover, a parallel proteomic study identified a set of deubiquitylating enzymes (DUBs) that progressively lose catalytic activity in the aging mouse brain due to cysteine thiol oxidation rather than changes in protein abundance, indicating that thiol redox imbalance may also broadly contribute to proteostasis decline during brain aging [26].

The biological consequences of this shift from persulfidation toward irreversible oxidation extend well beyond the regulation of individual enzymes. Emerging evidence indicates that cysteine post-translational modifications profoundly influence the biophysical behavior of proteins by regulating liquid–liquid phase separation (LLPS) and biomolecular condensation. Whereas persulfidation suppresses excessive protein condensation and preserves protein solubility, irreversible cysteine oxidation promotes the formation of dense condensates that progressively mature into pathological protein aggregates [21].

In this Review, we discuss the emerging role of protein persulfidation as a central regulator of proteostasis during aging and age-related disorders. We examine the molecular mechanisms governing protein persulfidation, its interplay with cysteine oxidation, LLPS, and biomolecular condensation, as well as recent advances in pharmacological, metabolic, and chemogenetic approaches aimed at restoring this endogenous mechanism. Finally, we highlight the therapeutic potential of targeting the H_2_S–persulfidation axis to preserve proteostasis, promote healthy aging, and delay the onset and progression of age-associated disorders.

## 2. Protein Persulfidation as an Emerging Regulator of Proteostasis

Proteome integrity is maintained by the proteostasis network (PN), a highly integrated, multi-compartmental system that coordinates protein synthesis, folding, and degradation to preserve protein homeostasis [27,28]. Together, these interconnected processes ensure that proteins acquire and maintain their native conformation while eliminating irreversibly damaged or misfolded species. Progressive decline of the PN is a fundamental hallmark of aging, resulting in the accumulation of oxidatively damaged and misfolded proteins that increasingly form pathological aggregates.

During the last decade, protein persulfidation has emerged as a key redox post-translational modification that protects reactive cysteine residues from irreversible oxidation while dynamically regulating protein function. Recent evidence indicates that the age-associated decline in protein persulfidation further compromises proteostasis by promoting oxidative protein damage, impairing cellular stress responses, and increasing susceptibility to pathological protein aggregation [7,25]. Mechanistically, protein persulfidation preserves proteostasis through several closely interconnected processes: (i) protecting reactive cysteine residues from irreversible hyperoxidation, (ii) regulating biomolecular condensation and preventing pathological protein aggregation, and (iii) maintaining the redox state of the chaperone and degradative machinery that constitutes the proteostasis network itself.

### 2.1. Protection Against Cysteine Hyperoxidation

**Figure 2:**
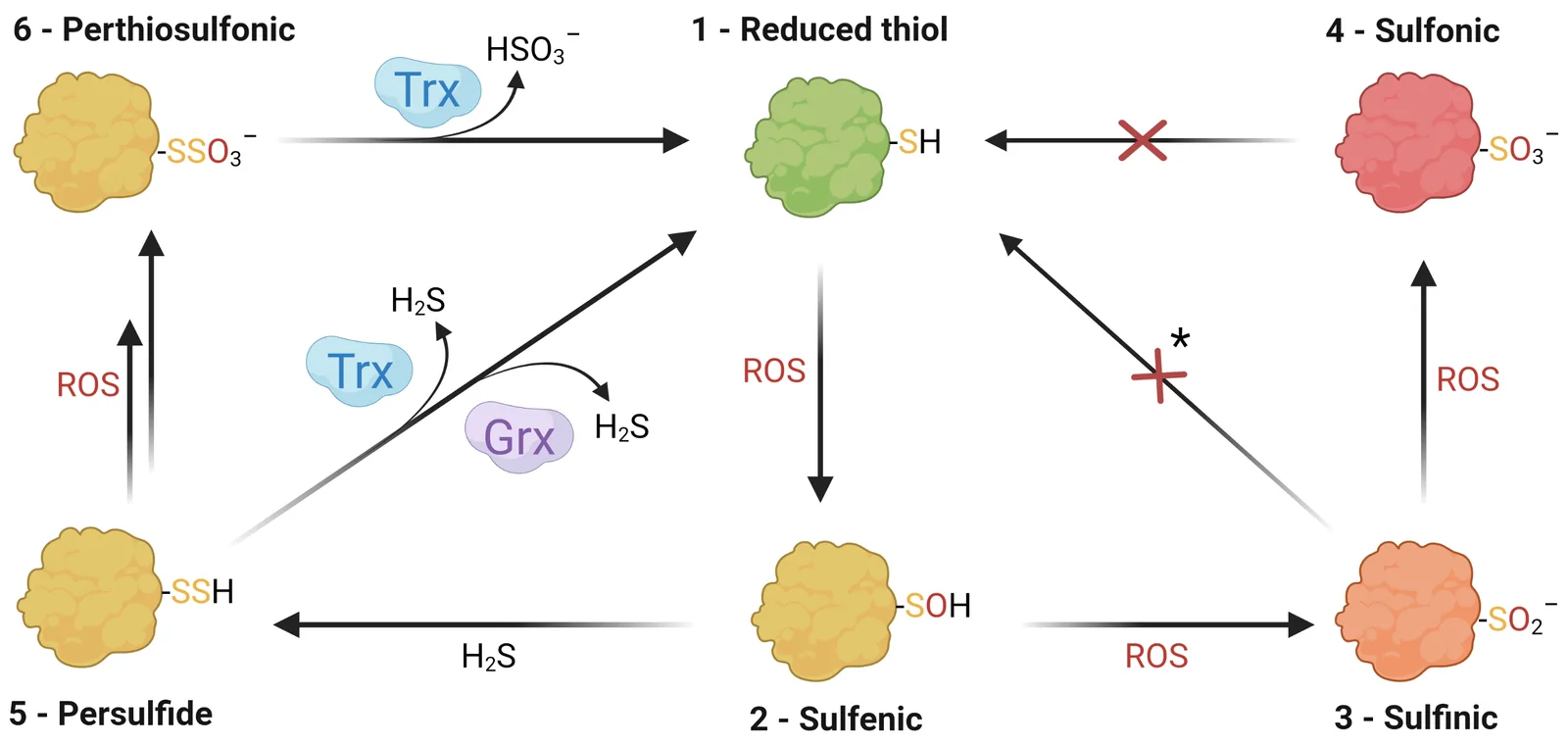
Persulfides act as a sacrificial redox buffer that prevents irreversible cysteine hyperoxidation. Direct oxidation of reduced protein thiols (1, PSH) proceeds through sulfenic acid (2, PSOH) and sulfinic acid (3, PSO_2_H) intermediates toward sulfonic acid (4, PSO_3_H), a stable and irreversible modification that permanently compromises protein structure and function. Reduction of the sulfinic acid back to the thiol does not occur under physiological conditions, with the exception of two-cysteine peroxiredoxins (*). Protein persulfides (5, PSSH) can be efficiently reduced back to the thiol by thioredoxin (Trx) and glutaredoxin (Grx). Note that oxidation of protein persulfides proceeds through analogous perthiosulfenic (PSSOH, not shown), perthiosulfinic (PSSO_2_H, not shown) and perthiosulfonic (6, PSSO_3_H) intermediates, via subsequent oxidation events. Unlike the thiol pathway, these persulfide oxidation products can be efficiently reduced back to the thiol by thioredoxin (Trx), so the additional sulfur atom absorbs oxidative modifications while keeping the reactive cysteine in a reversible, functional state.

The unique chemical properties of protein persulfides provide an elegant mechanism for preserving protein function under oxidative stress. Compared with thiols, persulfides are more nucleophilic and exhibit lower pKa values [29,30], enabling them to react preferentially with oxidizing species while remaining readily reducible by the thioredoxin [31] and glutaredoxin systems [32]. Consequently, protein persulfidation functions as a reversible redox shield that transiently modifies susceptible cysteine residues without permanently compromising their catalytic or structural roles (Figure 2).

Unlike direct oxidation of protein thiols, which can progress from sulfenic acid to sulfinic acid and ultimately irreversible sulfonic acid, oxidation of protein persulfides generates perthiosulfenic and perthiosulfinic intermediates that have been detected as oxidation products of different proteins, including papain, albumin, and glutathione peroxidase [30,33,34], which can be efficiently reduced back to the thiol (Figure 2). The additional sulfur atom therefore acts as a sacrificial oxidative buffer, preventing irreversible cysteine hyperoxidation while preserving protein activity during oxidative stress. This distinctive chemistry provides a mechanistic explanation for numerous observations demonstrating that increased protein persulfidation enhances cellular resistance to oxidative injury despite only modest changes in global ROS levels [25]. Moreover, by maintaining reactive cysteine residues in a reversible redox-active state, protein persulfidation preserves the structural and functional integrity of the proteome, thereby establishing the molecular foundation for its broader roles in proteostasis.

An important exception to the generally irreversible nature of cysteine hyperoxidation is found in typical 2-Cys peroxiredoxins (Prx1–4 in mammals) [35]. In these enzymes, the peroxidatic cysteine can be hyperoxidized to a sulfinic acid (PSO_2_H), which, unlike the sulfonic acid (PSO_3_H), can be enzymatically reduced back to the active thiol by the ATP-dependent reductase sulfiredoxin (Srx) [36,37]. This enzymatic repair pathway is restricted to this small group of thiol-dependent peroxidases and does not extend to sulfinylated cysteines in general.

### 2.2. Protein Persulfidation in the Proteostasis Network

#### 2.2.1. Chaperons

Recent evidence suggests that chaperone machinery is itself subject to age-dependent thiol remodelling. A detailed analysis of the recent proteomic datasets reported by Vignane et al. revealed that several components of the chaperone machinery, including HSP90α, HSP90β, HSP105/HSPH1, and the mitochondrial chaperone HSPA9, are endogenously persulfidated in the brain and undergo an age-dependent loss of this modification. This decline is most pronounced at middle age, followed by partial recovery at 18 months, reflecting the biphasic, non-linear behavior of the persulfidome as a whole [21]. Moreover, these chaperones show significant increased sulfenylation during aging and in CSE-deficient brain, where HSP105 is detected in its sulfonylated state.

Whether this remodeling impairs the heat shock response remains to be determined. Single cysteine modifications are sufficient to modulate chaperone activity, as shown for the inhibitory S-nitrosylation of HSP90, which inhibits its ATPase function [38]. On the contrary, HSF1 activation requires the redox-dependent modification of Cys35 and Cys105 for trimerization, DNA binding and induction of the HSP70 gene [39]. It is therefore attractive to propose that the age-associated loss of chaperone persulfidation contributes to the documented failure of the heat shock response in aged cells. However, the specific contribution of persulfidation, sulfenylation, and hyperoxidation to the regulation of the HSP90–HSF1–HSP70 axis remains largely unexplored, and further mechanistic studies will be required to test this hypothesis and establish the precise roles of these modifications in the age-associated decline of the heat shock response.

#### 2.2.2. The Proteasome

The degradative arm of the proteostasis network is similarly affected. The proteasome is the most strongly enriched pathway among proteins showing increased cysteine sulfenylation both during aging and in CSE-deficient brains, with the majority of detected subunits significantly affected, including all six ATPases of the 19S regulatory particle [21]. This is particularly relevant because cysteine oxidation of the 19S regulatory particle, rather than of the 20S catalytic core, is sufficient to inactivate the 26S proteasome without causing its disassembly [40], while mitochondrial oxidative stress can compromise the integrity of the 26S complex [41]. Notably, two subunits identified in the latter study as oxidized in response to mitochondrial ROS production, PSMC6/Rpt4 and PSMD12, are also significantly sulfenylated in the aging brain and in CSE-deficient animals, suggesting that the age-associated shift in cysteine redox state affects precisely those residues whose oxidation is known to impair proteasome function. Changes in proteasome persulfidation are more heterogeneous, with several α-subunits losing this modification during aging while others gain it. Thus, the recently available data indicate pronounced redox remodeling of the proteasome rather than a uniform loss of persulfidation. Given that proteasome activity declines in the aging brain and contributes to impaired proteostasis and protein aggregation [42], whether restoring specific persulfidation events can preserve proteasome function during aging remains an important question to be addressed in the field.

### 2.3. Protein Persulfidation as a Regulator of Biomolecular Condensation and Protein Aggregation

Many cellular processes are organized through biomolecular condensates, dynamic membrane-less compartments formed by LLPS [43,44,45]. These condensates concentrate proteins and nucleic acids to facilitate biochemical reactions while remaining highly dynamic and reversible under physiological conditions. Increasing evidence indicates that dysregulation of LLPS is a common feature of aging and numerous neurodegenerative disorders, in which normally dynamic condensates can progressively mature into solid-like assemblies and pathological protein aggregates [46,47].

Aberrant phase transitions have been reported for numerous aggregation-prone proteins implicated in neurodegenerative diseases and other age-related proteinopathies. Among the best-characterized examples is Tau, which undergoes LLPS that promotes fibrillization and neurofibrillary tangle formation in Alzheimer’s disease (AD) [48,49,50,51,52,53]. Similarly, α-synuclein forms biomolecular condensates that facilitate amyloid assembly and are associated with Lewy body pathology in Parkinson’s disease (PD) [47,48,54,55]. Expanded huntingtin also exhibits altered condensate dynamics that are thought to contribute to aggregate formation in Huntington’s disease (HD) [56,57,58].

RNA-binding proteins provide additional examples of the intimate relationship between biomolecular condensation and disease. In particular, TAR DNA-binding protein 43 (TDP-43) and fused in sarcoma (FUS) undergo LLPS under physiological conditions but can form persistent cytoplasmic condensates and pathological aggregates associated with amyotrophic lateral sclerosis (ALS) and frontotemporal dementia (FTD) [47,59,60,61,62,63,64,65,66,67,68]. Another illustrative example is heterogeneous nuclear ribonucleoprotein D-like isoform 2 (hnRNPDL-2), which undergoes LLPS and assembles into functional amyloid fibrils under physiological conditions [69,70,71]. Disease-causing mutations within exon 6, which encodes the C-terminal amyloid-forming low-complexity region of this protein, impair the formation of functional amyloid fibrils and cause autosomal-dominant limb-girdle muscular dystrophy D3, demonstrating that dysregulated phase transitions are associated not only to neurodegenerative disorders but also to neuromuscular disease [69,70,72].

Dysregulated biomolecular condensation has also been implicated in cancer. The tumor suppressor p53 undergoes LLPS to form dynamic condensates that can progressively mature into amyloid-like assemblies. Cancer-associated hotspot mutations have been found to accelerate this transition, linking altered condensate dynamics to the loss of p53 function and tumor progression, thereby extending the pathological relevance of LLPS beyond neurodegenerative disorders [73,74,75,76].

**Figure 3:**
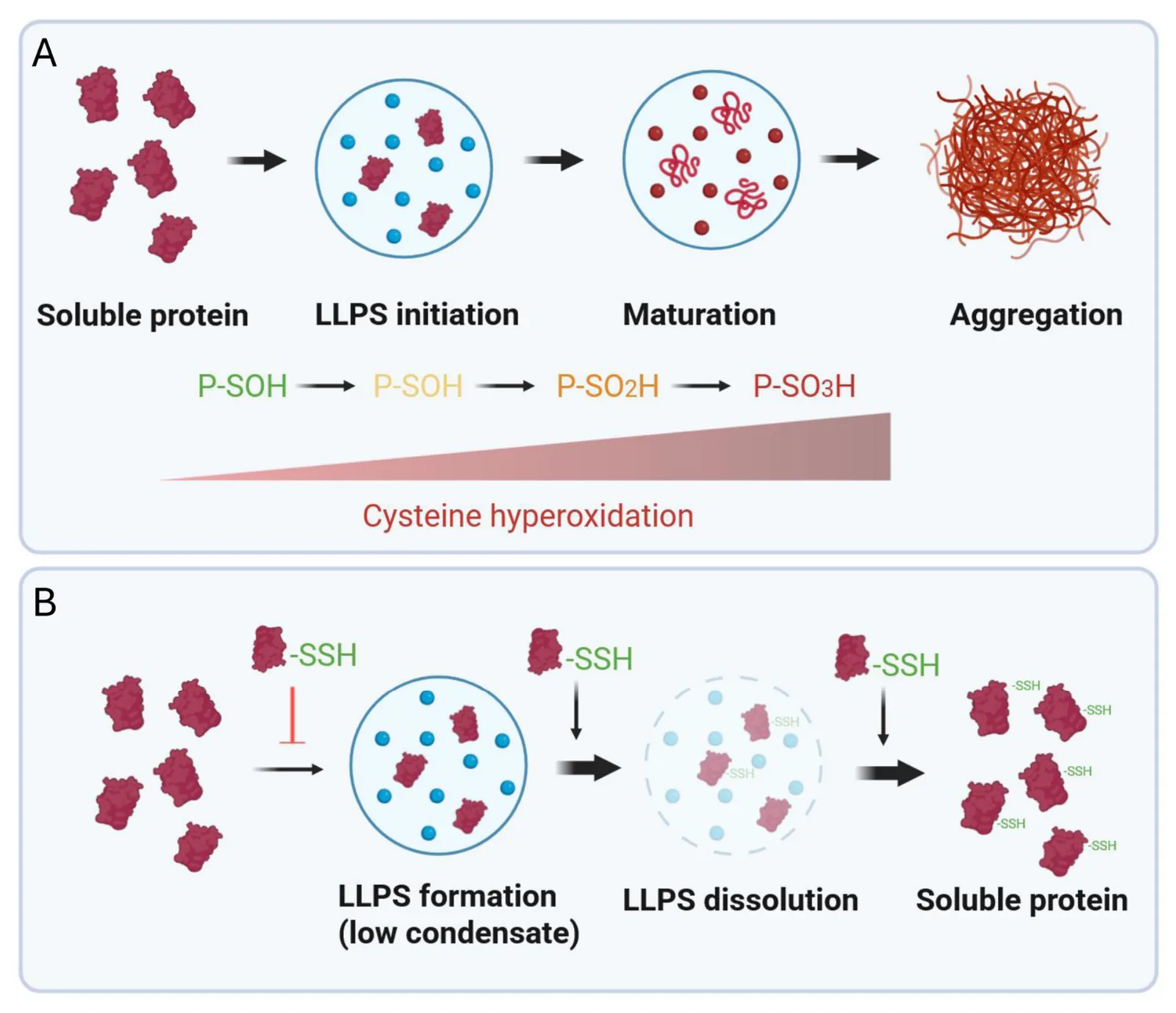
Protein persulfidation preserves proteostasis by preventing cysteine hyperoxidation and pathological biomolecular condensation. (**A**) Schematic representation of the cysteine sulfonylation-mediated aggregation model. Under physiological conditions, protein persulfidation maintains reactive cysteine residues in a reversible redox state, preserving protein solubility and preventing excessive liquid–liquid phase separation (LLPS). During aging, the progressive loss of persulfidation shifts cysteine modifications toward irreversible hyperoxidation, promoting LLPS by lowering the saturation threshold for biomolecular condensation and favoring the formation of dense protein condensates. Within these assemblies, reactive cysteine residues become increasingly susceptible to further oxidation, establishing a feed-forward cycle that drives condensate maturation into insoluble protein aggregates. (**B**) Conversely, restoration of protein persulfidation suppresses pathological phase separation, promotes dissolution of pre-existing condensates, limits irreversible cysteine oxidation, and preserves proteostasis by maintaining protein solubility and structural integrity.

A recent study identified cysteine redox chemistry as a previously unrecognized regulator of biomolecular condensation. Comprehensive proteomic analysis demonstrated that loss of endogenous persulfidation in CSE-deficient brains results in widespread redistribution of proteins into denser condensates with an increased propensity for oligomerization [21]. Multiple targets, including glycolytic enzymes, cytoskeletal proteins, RNA-binding proteins, and several regulators of neuronal function exhibited enhanced partitioning into high-density condensates, suggesting that protein persulfidation contributes to maintaining proteins in a soluble and functionally dynamic state [21]. Similar alterations were observed during physiological aging, linking impaired sulfide signaling to defective condensate homeostasis [21]. These observations are consistent with a cysteine sulfonylation-mediated aggregation mechanism, which provides a physicochemical framework connecting cysteine oxidation with proteostasis collapse (Figure 3).

According to this model, progressive cysteine hyperoxidation lowers the saturation threshold required for LLPS, facilitating the formation of dense biomolecular condensates. The increased local concentration of proteins within these condensates further promotes cysteine hyperoxidation, favoring persistent condensate stabilization and their subsequent maturation into aggregation-prone assemblies. This feed-forward mechanism links oxidative stress with defective condensate dynamics, protein aggregation, and progressive loss of proteostasis [21].

## 3. Loss of Persulfidation as a Driver of Brain Aging and Neurodegeneration

Aging progressively shifts the neuronal redox proteome from protective protein persulfidation toward irreversible cysteine oxidation, thereby disrupting proteostasis, neuronal function, and cellular resilience [21,23,25]. In this section, we summarize recent advances linking these changes to brain aging and neurodegeneration.

### 3.1. Age-Dependent Decline of Protein Persulfidation in the Aging Brain

A recent comprehensive chemoproteomic study provided the first proteome-wide characterization of age-dependent changes in protein persulfidation in the mammalian brain [21]. Using selective chemoproteomic labeling, the authors identified nearly 2000 endogenously persulfidated proteins in the mouse frontotemporal cortex, revealing that protein persulfidation is among the most abundant redox PTMs in the brain. Principal component analysis demonstrated extensive age-dependent remodeling of the persulfidome, with middle-aged and aged animals displaying distinct profiles compared with young mice. Approximately 40% of detected persulfidated proteins exhibited significant age-dependent changes, with the predominant trend corresponding to a global decline in persulfidation. Proteins undergoing age-dependent remodeling were enriched in pathways associated with neurodegeneration, proteostasis, synaptic transmission, and energy metabolism. Moreover, four of the six persulfidation clusters identified by trend analysis exhibited a progressive decline with age, and numerous proteins implicated in neurodegenerative diseases showed reduced persulfidation. Importantly, these changes occurred largely independently of protein abundance, as label-free quantitative proteomics performed on the same samples revealed only modest age-dependent differences in protein expression [21]. These findings indicate that aging is associated with extensive remodeling of the neuronal persulfidome that cannot be explained solely by changes in protein abundance, highlighting age-dependent regulation of protein persulfidation as an additional layer of proteome regulation. However, the complexity of age-associated proteome remodeling might also involve other post-translational modifications [77,78], changes in protein turnover and translation [79], and alterations in cellular metabolism [80], among other mechanisms.

Persulfidation did not decline uniformly throughout aging. Instead, chemoproteomic clustering identified a non-linear trajectory, with the most pronounced reduction occurring during middle age, followed by partial recovery for selected protein clusters in older animals [21]. This pattern resembles recently described non-linear molecular transitions observed across multiple omics datasets in aging humans and mice [81], suggesting that remodeling of the persulfidome may represent an early event in the aging process rather than simply a late consequence of accumulated oxidative damage.

### 3.2. Progressive Accumulation of Cysteine Oxidation to Sulfenic

The age-dependent decline in protein persulfidation is accompanied by a reciprocal increase in oxidative cysteine modifications. Analysis of the corresponding sulfenylome in the same brain samples demonstrated a progressive age-dependent accumulation of protein sulfenylation across virtually all cellular compartments [21]. Unlike persulfidation, which followed a complex non-linear pattern, sulfenylation increased progressively with age, indicating sustained oxidative remodeling throughout the lifespan.

Using the same chemoproteomic dataset, more than 1000 sulfenylated proteins were identified with significant enrichment in pathways involved in mitochondrial metabolism, protein folding, cytoskeletal organization, neuronal signaling, vesicle trafficking, and regulation of protein phosphorylation [21]. These oxidative modifications affected numerous proteins involved in synaptic function and neuronal maintenance, consistent with the progressive functional decline observed during physiological brain aging. Among these, several synaptic proteins that decline in abundance and function during aging, and that serve as classical markers of synaptic integrity such as synaptophysin, are functionally linked to CSE-derived H_2_S and persulfidation [82]. At the single protein level, synapsin 1, an abundant presynaptic phase separating protein, undergoes an age-dependent and CSE-dependent shift from persulfidation toward sulfenylation and sulfonylation that alters its phase behavior and solubility [21], directly connecting the loss of persulfidation to the presynaptic proteome. Lipofuscin represents a more complex age-associated marker, accumulating as heterogeneous aggregates of oxidized and cross-linked proteins and lipids as a consequence of impaired cellular clearance [83,84]. Although no direct mechanistic relationship between persulfidation and lipofuscin accumulation has been established, reduced persulfidation could potentially contribute to the oxidative damage and impaired proteostasis that favor its accumulation.

More importantly, comparison of the sulfenylome and persulfidome revealed a substantial overlap between proteins exhibiting increased oxidation and those undergoing loss of persulfidation. Approximately one-third of proteins displaying age-dependent remodeling exhibited both decreased persulfidation and increased sulfenylation, supporting a direct transition from protective to damaging cysteine modifications. Although sulfenic acid represents a reversible intermediate, persistent oxidative stress promotes its further oxidation to sulfinic and ultimately sulfonic acid (see Figure 2), modifications that are increasingly difficult—or, in the latter case, essentially impossible—to reverse.

### 3.3. Decline of Cystathionine γ-Lyase

Among the molecular events associated with the age-dependent decline in protein persulfidation, reduced endogenous H_2_S production has emerged as an important contributing factor. Accumulating evidence supports a prominent role for CSE-derived H_2_S in maintaining protein persulfidation in the aging brain [21,23]. Recent transcriptomic and metabolomic analyses by Vignane and colleagues demonstrated that *Cth*, the gene encoding CSE, progressively declines in the cerebral cortex during aging in both mice and humans, whereas expression of the other H_2_S-producing enzymes, CBS and MPST, remains relatively stable or even increases in certain brain regions [21]. Progressive decline in CSE expression was also accompanied by accumulation of its metabolic substrates, including cysteine and homocysteine, consistent with altered transsulfuration pathway activity during aging [21]. Furthermore, decreased CSE expression has been consistently reported in several neurodegenerative disorders, including Alzheimer’s disease, Huntington’s disease, and spinocerebellar ataxia, indicating that impaired endogenous sulfide production may represent a shared feature of several neurodegenerative conditions [85,86,87].

Evidence from CSE-deficient mouse models further supports an important contribution of CSE to the maintenance of the neuronal redox proteome [21]. Brains from CSE-deficient mice exhibit widespread loss of protein persulfidation together with marked increases in protein sulfenylation and irreversible sulfonylation, changes that closely resemble those observed during physiological aging. Remarkably, nearly 200 proteins display higher sulfenylation in young CSE-deficient mice than in naturally aged wild-type animals, indicating that CSE deficiency accelerates oxidative remodeling independently of chronological age. However, since CSE is an integral component of the transsulfuration pathway and contributes to sulfur amino acid metabolism beyond H_2_S production and protein persulfidation [88], the molecular phenotypes of CSE-deficient animals cannot be attributed exclusively to the loss of protein persulfidation and may also reflect broader metabolic consequences of CSE deficiency. Not surprisingly, CSE deficiency is also associated with pronounced alterations in protein homeostasis [21]. CSE-deficient mice exhibit widespread dysregulation of biomolecular condensation, enhanced liquid–liquid phase separation, spontaneous accumulation of protein aggregates during aging, and reduced lifespan [21]. Proteins involved in glycolysis, synaptic transmission, RNA metabolism, and stress responses display increased partitioning into dense condensates, consistent with a cysteine sulfonylation-mediated aggregation model described above. It is important to note that the relationship between altered biomolecular condensation and protein aggregation is protein- and context-dependent and does not necessarily follow a linear pathway, although altered condensates may undergo liquid-to-solid phase transitions, as demonstrated for several proteins associated with neurodegeneration, as discussed above and extensively reviewed elsewhere [43].

Beyond its effects on proteostasis, the decline in protein persulfidation also intersects with the regulation of inflammatory and immune signaling, which is particularly relevant given the neuroinflammatory changes associated with aging. In this context, two key regulators of the inflammatory response have emerged as direct persulfidation targets. Persulfidation of the NF-κB p65 subunit at Cys38 promotes its association with the coactivator ribosomal protein S3 and the transcription of anti-apoptotic and cytoprotective genes, a modification reported to decline with age [89]. Likewise, persulfidation of Keap1 at Cys151 promotes Nrf2 activation, coordinating antioxidant and anti-inflammatory responses and protecting against cellular senescence [90]. However, neither p65 nor Keap1 was detected in the aging-brain persulfidome, sulfenylome, or sulfonylome datasets [21], precluding a direct assessment of their age-associated redox remodeling. Nevertheless, declining endogenous H_2_S production during aging could potentially compromise persulfidation-dependent regulation of these pathways. Consistent with a broader in vivo impact of impaired H_2_S signaling, the genetic loss of CSE compromises blood–brain barrier integrity and promotes extravasation of peripheral immunoglobulin G into the brain parenchyma, features associated with the neuroinflammatory state that accompanies aging and neurodegeneration [82].

Overall, current evidence supports a model in which the age-dependent decline of CSE initiates a cascade of molecular events characterized by reduced protein persulfidation, progressive cysteine hyperoxidation, impaired biomolecular condensate dynamics, and ultimately proteostasis collapse. Rather than representing a secondary consequence of oxidative stress, the loss of endogenous protein persulfidation may represent an early contributor to brain aging and a potential therapeutic target for preventing or delaying neurodegeneration and other age-related disorders. The recognition of protein persulfidation as a central regulator of proteostasis has therefore shifted attention toward interventions capable of restoring this protective modification.

## 4. Strategies for Pharmacological Enhancement of Protein Persulfidation

Recognition of protein persulfidation as a central regulator of proteostasis has stimulated intense interest in therapeutic strategies aimed at restoring physiological sulfide signaling during aging. Because declining protein persulfidation appears to precede or accompany multiple hallmarks of aging, interventions that enhance sulfide biosynthesis or directly increase protein persulfidation represent promising therapeutic opportunities. Current approaches also include the modulation of sulfide-producing enzymes, administration of exogenous donors, dietary and metabolic interventions, and, more recently, chemogenetic strategies capable of generating H_2_S with high spatial and temporal precision. Despite their distinct mechanisms of action, these interventions share the common goal of restoring physiological protein persulfidation while minimizing off-target effects.

### 4.1. H_2_S-Producing Enzymes as Therapeutic Targets

Restoring endogenous H_2_S biosynthesis represents a physiologically attractive approach for enhancing protein persulfidation. In contrast to exogenous H_2_S donors, modulation of sulfide-producing enzymes could potentially preserve the spatial and temporal regulation of sulfur signaling by coupling H_2_S production to cellular metabolism [7]. However, therapeutic strategies targeting these enzymes remain largely unexplored.

Among the three principal mammalian H_2_S-producing enzymes, CSE has received particular attention in the context of brain aging because recent studies have documented an age-dependent decline in its expression together with alterations in protein persulfidation and cysteine oxidation [7,11,86]. CBS and MPST also contribute to maintaining protein persulfidation in a tissue-dependent manner. CBS is the predominant H_2_S-producing enzyme in the central nervous system and additionally regulates one-carbon metabolism and homocysteine homeostasis, whereas MPST contributes to mitochondrial sulfur metabolism and the generation of reactive sulfur species [10,12,13]. Although these enzymes have been implicated in cellular redox regulation, their specific roles in preserving protein persulfidation during aging remain poorly understood, and selective pharmacological modulators are still limited. A further limitation is that the relative contributions of CSE, CBS, and MPST to H_2_S production and protein persulfidation vary across tissues, cell types, and subcellular compartments, reflecting their distinct expression patterns, intracellular localization, and metabolic functions [88].

Current evidence provides a biological rationale for exploring the modulation of sulfide-producing enzymes as a means to restore protein persulfidation. However, this concept remains largely hypothetical, as no studies have yet demonstrated that pharmacological activation of CSE, CBS, or MPST effectively restores protein persulfidation or delays aging and age-related diseases. Future work will therefore be required to determine whether enzyme-targeted approaches can be translated into effective therapeutic strategies.

### 4.2. Pharmacological Strategies to Enhance Protein Persulfidation

Beyond enhancing endogenous H_2_S biosynthesis, increasing attention has been devoted to pharmacological approaches aimed at promoting protein persulfidation. These include fast- and slow-releasing H_2_S donors, hybrid H_2_S-releasing drugs, sulfur-containing natural products, and emerging chemogenetic technologies. Although mechanistically diverse, these approaches share the common objective of increasing intracellular sulfide availability while avoiding the supraphysiological concentrations associated with conventional sulfide salts. Table 1 summarizes the best-characterized compounds and interventions reported to enhance protein persulfidation.

Among the most widely used experimental sources of H_2_S are inorganic sulfide salts, such as sodium hydrosulfide (NaHS) and sodium sulfide (Na_2_S), which rapidly release H_2_S upon dissolution [7,30]. Although these compounds have been instrumental in defining H_2_S biology, their rapid release kinetics poorly mimic endogenous sulfide production, resulting in non-physiological concentration bursts, limited tissue specificity, and potential off-target effects.

Consequently, newer generations of slow-releasing donors, such as GYY4137, AP39, AP123, and related compounds, have been developed to achieve more sustained sulfide delivery and improved pharmacokinetic profiles [92,93,94]. Compared with bolus sulfide salts, GYY4137 has proven useful as a tool to demonstrate that pharmacologically induced persulfidation can reproduce effects otherwise only inferred from endogenous H_2_S signaling. In primary hippocampal neurons, pretreatment with GYY4137 accelerated KCl-triggered synaptic vesicle release, consistent with H_2_S-induced persulfidation driving the dispersion of synapsin-1 from presynaptic condensates. In cellular models of impaired CSE activity, including CSE-/- fibroblasts and striatal cells expressing mutant huntingtin (STHdhQ111/Q111), GYY4137 treatment markedly reduced the size of arsenite-induced G3BP2 stress granules, mirroring the dissolution seen with direct H_2_S addition and confirming that slow-releasing donors can rescue a persulfidation deficit at the level of biomolecular condensates [21]. By comparison, the mitochondria-targeted donors AP39 and AP123 achieve considerably stronger persulfidation per mole than GYY4137, since their triphenylphosphonium moiety concentrates H_2_S release directly within the mitochondrial matrix rather than releasing it diffusely in the cytosol. Even at nanomolar concentrations, AP39 has been reported to increase intracellular persulfide levels more strongly than any other H_2_S donor tested, and AP123 shows comparable protective effects against oxidative stress in renal epithelial cells [31,93].

Another promising strategy involves dietary and metabolic interventions, which provide an alternative means of enhancing endogenous persulfidation. Specifically, sulfur amino acid restriction, methionine restriction, and caloric restriction consistently increase transsulfuration pathway activity and endogenous H_2_S production, effects that appear to contribute to the health benefits of these interventions [25,97,99].

Chemogenetic technologies have introduced a fundamentally different approach to manipulating intracellular sulfide signaling. The H_2_SWITCH platform utilizes *Salmonella typhimurium* D-cysteine desulfhydrase to catalyze selective intracellular H_2_S production following administration of D-cysteine, enabling spatiotemporally controlled sulfide generation with minimal interference from endogenous metabolism [98]. Unlike conventional donors, this system permits precise control over the location and timing of H_2_S production, providing a valuable platform for investigating persulfidation-dependent signaling and potentially enabling future targeted therapeutic applications [98].

More recently, ergothioneine has emerged as a metabolic modulator that enhances CSE-dependent H_2_S production and protein persulfidation without acting as a prototypical sulfide donor. ET stimulates physiological sulfide biosynthesis through the transsulfuration pathway, providing proof-of-concept that pharmacological restoration of endogenous protein persulfidation may improve healthy aging. The underlying mechanisms and preclinical evidence are discussed in the following section [91].

### 4.3. Safety Considerations and Limitations of H_2_S-Based Therapies

H_2_S-based interventions raise several clinically relevant safety considerations. Because CSE-derived H_2_S acts as an endogenous vasorelaxant, systemic administration of H_2_S donors can affect vascular tone and blood pressure, consistent with the hypertensive phenotype observed in CSE-deficient mice [100]. H_2_S signaling and protein persulfidation also operate extensively in peripheral tissues, including in the regulation of renal blood flow [101]. Thus, when considering therapeutic applications targeting the nervous system, the limited tissue selectivity of currently available H_2_S donors should be taken into account, as systemic exposure may produce off-target effects, including persulfidation in peripheral tissues. Moreover, H_2_S exhibits concentration-dependent, biphasic biological effects. Whereas low concentrations can exert cytoprotective effects, high concentrations inhibit mitochondrial cytochrome *c* oxidase, suppress oxidative phosphorylation, and may promote pro-oxidant effects [102,103]. These risks are particularly relevant for donors with non-physiological release kinetics or limited spatial and temporal control over H_2_S delivery (Table 1). All in all, these limitations illustrate a central principle of redox-based therapeutic strategies: successful clinical translation requires careful control of dose, tissue and subcellular localization, and biological context [104,105].

## 5. Ergothioneine as a Proof-of-Concept for Persulfidation Therapy

Ergothioneine (ET) is a naturally occurring sulfur-containing amino acid derivative synthesized by fungi and certain bacteria and acquired in mammals exclusively through the diet via the organic cation transporter SLC22A4 [91,106]. Although originally recognized as a potent antioxidant owing to its remarkable chemical stability and accumulation in tissues exposed to oxidative stress, its molecular mechanism of action remained unclear for many years [107,108].

### 5.1. Ergothioneine Stimulates CSE-Dependent Persulfidation

**Figure 4:**
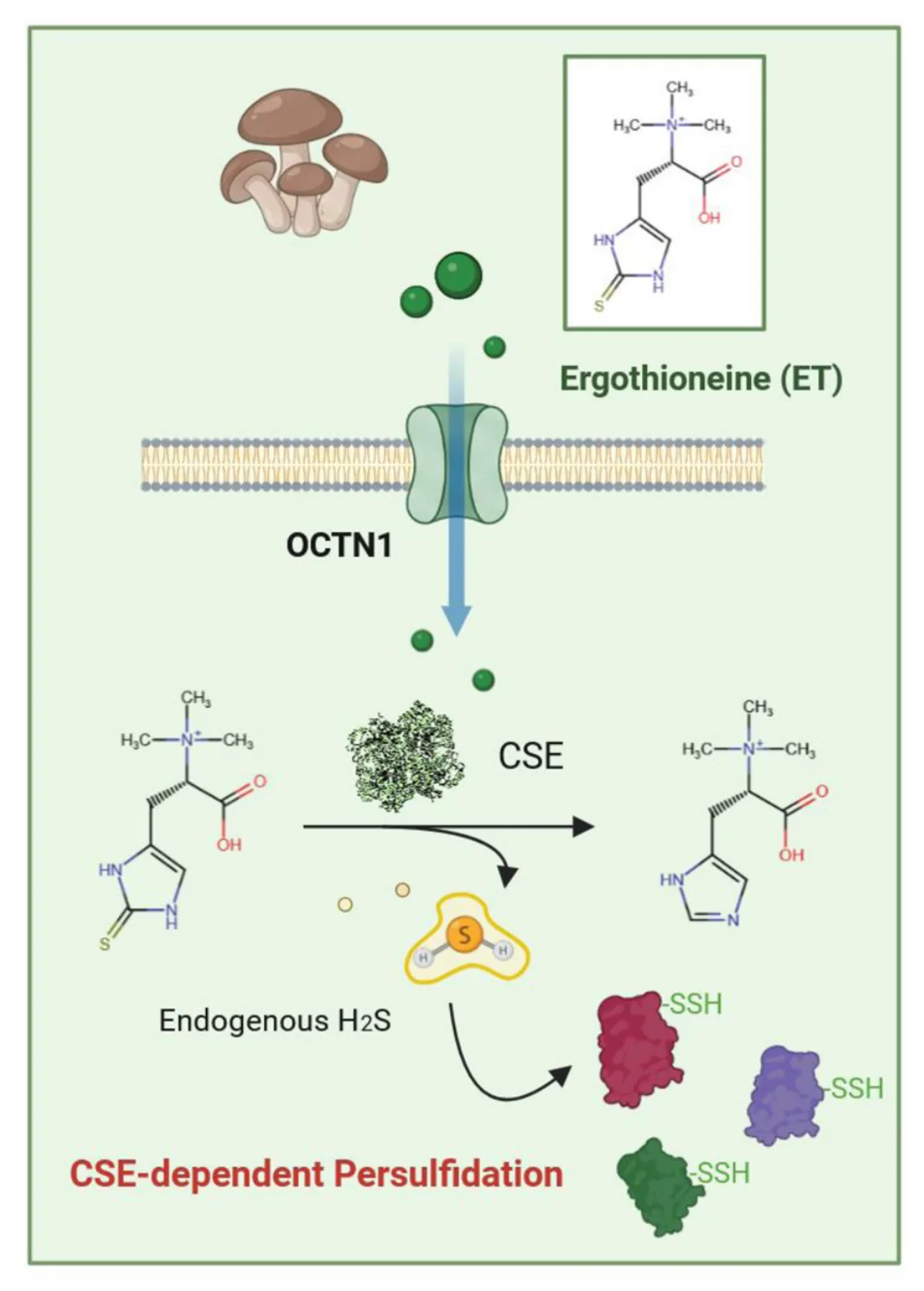
Ergothioneine restores protein persulfidation and promotes healthy aging through activation of the CSE–H_2_S signaling axis. Dietary ergothioneine is transported into cells primarily through the organic cation transporter OCTN1 (SLC22A4). ET functions as an alternative substrate for cystathionine γ-lyase (CSE), stimulating endogenous hydrogen sulfide (H_2_S) production without directly acting as a sulfide donor. (B) Increased endogenous H_2_S enhances global protein persulfidation of proteins involved in mitochondrial metabolism, energy production, protein quality control, stress responses, and redox signaling. Among these targets, cytosolic glycerol-3-phosphate dehydrogenase (cGPDH) undergoes persulfidation at regulatory cysteine residues, resulting in increased enzymatic activity. Persulfidation-dependent activation of cGPDH enhances glycerol phosphate shuttle activity, improves cellular redox balance, increases NAD^+^ availability, and supports mitochondrial oxidative phosphorylation and ATP production. These metabolic adaptations improve mitochondrial function while reducing oxidative stress and preserving cellular homeostasis.

A recent study has revised this paradigm by demonstrating that ET functions as an alternative substrate for CSE, thereby stimulating endogenous H_2_S production [91]. In aged *Caenorhabditis elegans* and rats, ET treatment enhanced protein persulfidation, promoted persulfidation-dependent activation of cGPDH, increased NAD^+^ biosynthesis, and significantly improved lifespan, healthspan, muscle performance, and stress resistance [91]. Unlike rapidly releasing H_2_S donors, which frequently generate transient supraphysiological sulfide concentrations, ET promotes sustained endogenous H_2_S production coupled to cellular metabolism. Importantly, ET-induced H_2_S production was abolished by pharmacological inhibition or genetic deletion of CSE, demonstrating that its biological effects depend on stimulation of endogenous sulfide biosynthesis rather than direct antioxidant activity (Figure 4). Consistent with this mechanism, ET induces a robust increase in global protein persulfidation. Quantitative persulfidome analysis identified hundreds of proteins exhibiting increased persulfidation following ET treatment, including proteins involved in mitochondrial metabolism, glycolysis, oxidative phosphorylation, protein folding, cytoskeletal organization, and cellular stress responses [91]. These findings demonstrate that ET promotes widespread remodeling of the persulfidome, extending its effects well beyond individual protein targets.

In summary, this recent study has provided direct evidence that pharmacological stimulation of endogenous protein persulfidation can improve physiological function during aging, establishing ET as a proof-of-concept therapeutic strategy targeting the H_2_S–protein persulfidation axis [91].

### 5.2. Persulfidation of cGPDH Links H_2_S Signaling to NAD^+^ Metabolism

As pointed above, one of the most important mechanistic insights arising from studies of ET is the identification of cGPDH as a critical persulfidation target linking H_2_S signaling with cellular metabolism. cGPDH catalyzes the reversible conversion of dihydroxyacetone phosphate to glycerol-3-phosphate while reducing NAD^+^ to NADH, thereby participating in the glycerol phosphate shuttle and coordinating cytosolic and mitochondrial redox balance [109]. Not surprisingly, proteomic analyses demonstrated that ET markedly increases persulfidation of cGPDH at specific regulatory cysteine residues, resulting in enhanced enzymatic activity [91]. In this study, structural modeling further suggested that persulfidation induces subtle conformational changes that favor substrate binding and catalytic efficiency, providing a direct molecular mechanism by which H_2_S regulates metabolic enzyme activity. More importantly, mutation of the target cysteine residue abolished both persulfidation and enzyme activation, confirming that this modification is required for the metabolic effects of ET.

Enhanced cGPDH activity was also accompanied by increased intracellular NAD^+^ levels and improved NAD^+^/NADH homeostasis in multiple experimental models. Because NAD^+^ availability declines during aging and contributes to mitochondrial dysfunction, impaired DNA repair, and reduced activity of NAD^+^-dependent enzymes such as sirtuins, restoration of NAD^+^ metabolism represents an attractive strategy for promoting healthy aging [91,110]. Together, these findings identify protein persulfidation as a previously unrecognized regulator of NAD^+^ metabolism and establish cGPDH as a molecular link connecting sulfur metabolism with cellular bioenergetics.

### 5.3. Benefits of Ergothioneine Supplementation in Aging Organisms

The metabolic effects of ET translate into significant physiological improvements in animal models of aging. In *Caenorhabditis elegans*, dietary ET supplementation significantly extended both lifespan and healthspan while enhancing locomotor activity, stress resistance, and maintenance of physiological function during aging [91]. These beneficial effects were abolished in CSE-deficient worms, demonstrating that longevity depends on endogenous H_2_S production and protein persulfidation rather than direct antioxidant activity [91].

Similarly, dietary ET administration to naturally aged rats produced marked improvements in multiple parameters associated with healthy aging. ET-treated animals exhibited preservation of skeletal muscle mass, increased muscle fiber vascularization, enhanced endurance capacity, and improved exercise performance compared with untreated age-matched controls [91]. These physiological improvements were accompanied by increased global protein persulfidation and enhanced cGPDH activity, supporting a mechanistic relationship between restoration of protein persulfidation and improved tissue function.

ET supplementation did not simply attenuate oxidative damage but promoted coordinated improvements in mitochondrial metabolism, proteostasis, and tissue function, highlighting the multifaceted role of protein persulfidation in maintaining cellular resilience during aging. These findings distinguish ET from classical antioxidant therapies, which generally act by scavenging reactive oxygen species without restoring endogenous redox signaling pathways [91,111,112].

Viewed against the safety considerations discussed above, ergothioneine raises particular interest because it does not act as a conventional sulfide donor but instead stimulates endogenous, CSE-dependent H_2_S production coupled to cellular metabolism. This could avoid the supraphysiological sulfide spikes, off target persulfidation, and biphasic toxicity associated with bolus donors. Moreover, this mechanism depends on CSE, making its effect inherently tissue selective depending on the level of the enzyme expressed in each tissue. This same dependence, however, represents its main limitation, since CSE expression itself declines with aging, so that the substrate-based stimulation of H_2_S production would progressively lose efficacy and ultimately fail in the tissues where the enzyme is most absent. Its efficacy and safety in humans therefore remain established.

## 6. Conclusions and Future Perspectives

Over the past decade, protein persulfidation has emerged from a relatively overlooked redox post-translational modification to an important regulatory mechanism implicated in cellular homeostasis that links H_2_S signaling with proteostasis, metabolism, and healthy aging. Advances in quantitative chemoproteomics have transformed our understanding of the persulfidome, revealing that thousands of proteins undergo dynamic persulfidation in vivo and that this modification is extensively remodeled during aging [21,23]. These seminal studies have identifed protein persulfidation as a pervasive regulatory mechanism controlling mitochondrial function, protein quality control, cellular signaling, stress responses, and metabolic adaptation [7,15,21].

One of the most important conceptual advances arising from recent work is the recognition that aging is accompanied not simply by increased oxidative stress but by progressive disruption of cysteine redox homeostasis. The age-dependent decline in protein persulfidation, together with the accumulation of irreversible oxidative cysteine modifications, provides a mechanistic framework potentially linking impaired H_2_S signaling to loss of protein function, proteostasis collapse, and cellular dysfunction [21]. Moreover, the discovery that protein persulfidation can modulate biomolecular condensation and suppress pathological phase transitions has substantially broadened the biological significance of this modification. By integrating cysteine redox chemistry with condensate biology, the cysteine sulfonylation-mediated aggregation model offers a plausible explanation for how oxidative imbalance may contribute to protein aggregation during aging and neurodegeneration [21].

These mechanistic advances have also opened new therapeutic opportunities. Rather than simply scavenging reactive oxygen species after oxidative damage has occurred, strategies that restore protein persulfidation aim to preserve physiological redox signaling and maintain protein function before irreversible damage develops. Pharmacological activation of sulfide-producing enzymes, slow-releasing H_2_S donors, dietary and metabolic interventions, and emerging chemogenetic approaches all represent promising strategies for enhancing protein persulfidation [7,91,98]. Among these, ET provides proof-of-concept in preclinical models that stimulation of endogenous sulfide biosynthesis can restore the persulfidome, improve mitochondrial metabolism, enhance NAD^+^ homeostasis, and promote healthspan [23,91].

Despite these advances, several important challenges must be addressed before persulfidation-targeted therapies can be translated into clinical practice. A major priority will be identifying the specific persulfidation events that determine cellular resilience. Although thousands of modified proteins have now been identified, it remains unclear whether the beneficial effects of H_2_S result from coordinated remodeling of the persulfidome or from modulation of a relatively small number of critical regulatory proteins [21]. In particular, the ubiquitin–proteasome system, largely responsible for protein turnover in the cell and itself extensively sulfenylated during aging, has been shown to be particularly sensitive to inactivation by cysteine oxidation [26,40,113]. Moreover, whether the redox-active cysteines of HSF1 and HSP90 are persulfidation targets, and whether their modification influences HSP90–HSF1 complex dynamics and the induction of the heat shock response, remains unexplored and represents a matter of future investigation. The same applies to the functional consequences of proteasome subunit oxidation, and to whether restoring persulfidation preserves proteasome activity in the aging brain. Further characterization of these functionally relevant targets will be essential for developing precise therapeutic interventions.

Achieving the selective modulation of protein persulfidation also remains a significant challenge. Current sulfide donors generally increase intracellular sulfide concentrations without tissue or protein specificity, increasing the potential for off-target effects. Future strategies should focus on selectively enhancing protein persulfidation within defined tissues, cell types, subcellular compartments, or even individual proteins [98]. The safety profile will also be decisive, given the biphasic pharmacology of H_2_S and the potential for mitochondrial inhibition and pro-oxidant effects at excessive or poorly targeted exposures [104,105]. Moreover, strategies relying on endogenous enzymatic capacity, such as substrate-based stimulation of CSE, may be constrained by tissue- and cell-specific CSE expression and activity, as well as by its age-dependent decline. Future advances in targeted delivery, organelle-directed donors, enzyme-selective modulators, and chemogenetic technologies may provide the precision required for clinical translation.

Equally important is the development of clinically applicable biomarkers. Although quantitative chemoproteomic methods have revolutionized experimental studies of protein persulfidation, they remain technically demanding and unsuitable for routine clinical use [22]. Robust and minimally invasive biomarkers capable of monitoring protein persulfidation in patients will be essential for diagnosis, patient stratification, pharmacodynamic assessment, and evaluation of therapeutic responses. Integration of persulfidomics with metabolomics, redox proteomics, and artificial intelligence-assisted biomarker discovery may accelerate this process.

Several important questions remain to be addressed. The molecular determinants governing the selectivity of protein persulfidation are still incompletely understood, including the structural landscape and the particular contributions of local sulfur metabolism and subcellular compartmentalization in regulating this process. Likewise, the interplay between protein persulfidation and other cysteine PTMs—including S-nitrosylation, glutathionylation, sulfenylation, and lipid-derived modifications—remains largely unexplored. Addressing these questions will be essential for understanding how multiple redox signals are integrated to regulate protein function under physiological and pathological conditions. Furthermore, although most mechanistic studies have focused on the nervous system, accumulating evidence indicates that protein persulfidation is involved in a variety of age-associated processes across multiple organs, including skeletal muscle, the cardiovascular system, liver, kidney, immune cells, and stem cells, suggesting that its contribution to organismal aging may extend beyond the nervous system [91].

Taken together, protein persulfidation has emerged as an important regulator of proteostasis and cellular resilience, linking sulfur metabolism, mitochondrial function, redox homeostasis, and protein quality control. Continued advances in persulfidomics, redox biology, and precision therapeutic approaches are expanding our understanding of this regulatory mechanism and its potential relevance to age-related disorders. Restoring physiological protein persulfidation therefore represents a compelling therapeutic opportunity to preserve proteome integrity, maintain tissue function, and promote healthy aging.

## Figures and Tables

**Table 1:** Overview of pharmacological and metabolic strategies reported to enhance protein persulfidation.

Compound Name	Class/Origin	Mechanism of Action	Evidence	Limitations	Ref.
Ergothioneine (ET)	Fungi-derived natural sulfur metabolite	Stimulates CSE-dependent endogenous H_2_S production, increasing global protein persulfidation	Cell culture, *C. elegans*, aged rats	Human efficacy not yet established. Needs CSE that decays with aging	[91]
NaHS/Na_2_S	Inorganic sulfide salts	Rapid dissociation to HS-/H_2_S in aqueous solution; reacts with protein disulfides and sulfenic acids to form persulfide. Requires an oxidized cysteine acceptor rather than reacting directly with a reduced thiol.	Extensively used in vitro and in rodent models of ischemia–reperfusion, inflammation and neuroprotection	Bolus, non-physiological release kinetics. Stock solutions often contaminated with reactive inorganic polysulfides. plus stronger solution alkalinity that complicates dosing (Na_2_S)	[7,30]
GYY4137	Slow releasing phosphorodithioate H_2_S donor	pH dependent hydrolysis releases H_2_S gradually, more closely approximating endogenous production than sulfide salts; downstream persulfidation of targets such as Keap1 and the NF-kB p65 subunit has been reported	Anti-inflammatory, anti-atherosclerotic and vasodilatory effects in cell and rodent models	Hydrolysis mechanism and precise H_2_S release kinetics still debated; phosphorus containing hydrolysis products of unclear biological activity	[7,92]
AP39/AP123	Mitochondria-targeted H_2_S donors (dithiolethione conjugated to triphenylphosphonium)	Lipophilic cation drives accumulation in the mitochondrial matrix, allowing local H_2_S release. Produces markedly greater increases in intracellular persulfidation per mole than untargeted donors	Cytoprotection in hyperglycemic injury and oxidative stress models; strong increase in cellular persulfide levels even at nanomolar concentrations	Limited pharmacokinetic characterization; potential off-target effects from the lipophilic phosphonium cation at higher doses	[31,93,94]
SG1002	Sulfur amino acid based oral H_2_S prodrug	Slow release of H_2_S after oral administration and metabolic processing, increasing circulating H_2_S and downstream NO bioavailability	Phase I clinical trial in heart failure patients showed increased plasma H_2_S and improved NO bioavailability with acceptable tolerability	Effect on protein persulfidation was not directly measured in the clinical study; release mechanism only partially characterized	[95]
Diallyl trisulfide (DATS)	Garlic-derived organic polysulfide	Reacts with glutathione and other cellular thiols to generate allyl persulfide and glutathione persulfide (GSSH), which can transpersulfidate protein targets and release H_2_S	Cardioprotective, antihypertensive and antiproliferative effects linked in part to persulfidation of targets such as Keap1	Chemical instability, difficulty standardizing dose, and possible contamination with reactive inorganic polysulfides	[24,96]
Diallyl disulfide (DADS)	Garlic-derived organic disulfide, metabolic precursor of DATS	Same thiol mediated decomposition pathway as DATS, generating allyl persulfide and H_2_S, generally with lower potency	Cardiovascular and chemopreventive effects broadly similar to DATS	Same instability and standardization issues as DATS	[24,96]
**Tool/Intervention**	**Class/Origin**	**Mechanism of action**	**Evidence**	**Limitations**	**Ref.**
Methionine restriction	Dietary and nutritional intervention	Reduced methionine intake reroutes flux through the transsulfuration pathway and has been linked to increased hepatic H_2_S production, indirectly supporting persulfide formation	Hine et al. 2015 showed that many metabolic benefits of dietary restriction in mice require endogenous H_2_S production via the transsulfuration enzyme CGL/CSE	Sustained methionine restriction has limited translational feasibility in humans	[97]
H_2_SWITCH	Chemogenetic enzymatic tool (bacterial D-cysteine desulfhydrase, stDCyD)	Enzymatic alpha,beta elimination of D-cysteine generates H_2_S in a dose tunable, enantioselective and redox orthogonal manner inside living cells, enabling controlled study of persulfidation independent of conventional donor chemistry	Validated as a precision tool for spatiotemporal control of intracellular H_2_S flux in cell based systems	Requires genetic delivery of a bacterial enzyme; a research tool rather than a therapeutic strategy; not yet tested in aging or in vivo disease models	[98]
Caloric restriction	Broader dietary and metabolic intervention	Proposed to increase H_2_S production through overlapping mechanisms with sulfur amino acid restriction, and increase in CSE expression.	Direct, persulfidation increases with caloric restriction, and extends lifespan	Sustained, life-long caloric restriction has limited translational feasibility in humans.	[25,97,98]

## Data Availability

No new data were created or analyzed in this study. Data sharing is not applicable to this article.

## References

[1] Gladyshev V.N. (2016). Aging: Progressive decline in fitness due to the rising deleteriome adjusted by genetic, environmental, and stochastic processes. Aging Cell.

[2] Van Dam L., Dansen T.B. (2020). Cross-talk between redox signalling and protein aggregation. Biochem. Soc. Trans..

[3] Finkel T. (2015). The metabolic regulation of aging. Nat. Med..

[4] Holmstrom K.M., Finkel T. (2014). Cellular mechanisms and physiological consequences of redox-dependent signalling. Nat. Rev. Mol. Cell Biol..

[5] Finkel T., Holbrook N.J. (2000). Oxidants, oxidative stress and the biology of ageing. Nature.

[6] Jones D.P., Sies H. (2015). The Redox Code. Antioxid. Redox Signal..

[7] Filipovic M.R., Zivanovic J., Alvarez B., Banerjee R. (2018). Chemical Biology of H_2_S Signaling through Persulfidation. Chem. Rev..

[8] Mustafa A.K., Gadalla M.M., Snyder S.H. (2009). Signaling by gasotransmitters. Sci. Signal..

[9] Mustafa A.K., Gadalla M.M., Sen N., Kim S., Mu W., Gazi S.K., Barrow R.K., Yang G., Wang R., Snyder S.H. (2009). H_2_S signals through protein S-sulfhydration. Sci. Signal..

[10] Kabil O., Banerjee R. (2014). Enzymology of H_2_S biogenesis, decay and signaling. Antioxid. Redox Signal..

[11] Chiku T., Padovani D., Zhu W., Singh S., Vitvitsky V., Banerjee R. (2009). H_2_S biogenesis by human cystathionine gamma-lyase leads to the novel sulfur metabolites lanthionine and homolanthionine and is responsive to the grade of hyperhomocysteinemia. J. Biol. Chem..

[12] Kabil O., Vitvitsky V., Xie P., Banerjee R. (2011). The quantitative significance of the transsulfuration enzymes for H_2_S production in murine tissues. Antioxid. Redox Signal..

[13] Singh S., Padovani D., Leslie R.A., Chiku T., Banerjee R. (2009). Relative contributions of cystathionine beta-synthase and gamma-cystathionase to H_2_S biogenesis via alternative trans-sulfuration reactions. J. Biol. Chem..

[14] Luo S., Kong C., Ye D., Liu X., Wang Y., Meng G., Han Y., Xie L., Ji Y. (2023). Protein Persulfidation: Recent Progress and Future Directions. Antioxid. Redox Signal..

[15] Paulsen C.E., Carroll K.S. (2013). Cysteine-mediated redox signaling: Chemistry, biology, and tools for discovery. Chem. Rev..

[16] Cuevasanta E., Benchoam D., Semelak J.A., Moller M.N., Zeida A., Trujillo M., Alvarez B., Estrin D.A. (2022). Possible molecular basis of the biochemical effects of cysteine-derived persulfides. Front. Mol. Biosci..

[17] Vignane T., Filipovic M.R. (2023). Emerging Chemical Biology of Protein Persulfidation. Antioxid. Redox Signal..

[18] Vignane T., Filipovic M.R. (2023). Emerging Chemical Biology of Protein Persulfidation. Antioxid. Redox Signal..

[19] Peralta D., Bronowska A.K., Morgan B., Doka E., Van Laer K., Nagy P., Grater F., Dick T.P. (2015). A proton relay enhances H_2_O_2_ sensitivity of GAPDH to facilitate metabolic adaptation. Nat. Chem. Biol..

[20] Kuehne A., Emmert H., Soehle J., Winnefeld M., Fischer F., Wenck H., Gallinat S., Terstegen L., Lucius R., Hildebrand J. (2015). Acute Activation of Oxidative Pentose Phosphate Pathway as First-Line Response to Oxidative Stress in Human Skin Cells. Mol. Cell.

[21] Vignane T., Hugo M., Hoffmann C., Reichert P., Katsouda A., D’Andrea D., Wang H., Tromm J.V., Miler M., Comas F. (2026). Protein thiol alterations drive pathologic liquid-liquid phase separation in the aging brain. Nat. Struct. Mol. Biol..

[22] Anjo S.I., He Z., Hussain Z., Farooq A., McIntyre A., Laughton C.A., Carvalho A.N., Finelli M.J. (2024). Protein Oxidative Modifications in Neurodegenerative Diseases: From Advances in Detection and Modelling to Their Use as Disease Biomarkers. Antioxidants.

[23] Petrovic D., Kouroussis E., Vignane T., Filipovic M.R. (2021). The Role of Protein Persulfidation in Brain Aging and Neurodegeneration. Front. Aging Neurosci..

[24] Ida T., Sawa T., Ihara H., Tsuchiya Y., Watanabe Y., Kumagai Y., Suematsu M., Motohashi H., Fujii S., Matsunaga T. (2014). Reactive cysteine persulfides and S-polythiolation regulate oxidative stress and redox signaling. Proc. Natl. Acad. Sci. USA.

[25] Zivanovic J., Kouroussis E., Kohl J.B., Adhikari B., Bursac B., Schott-Roux S., Petrovic D., Miljkovic J.L., Thomas-Lopez D., Jung Y. (2019). Selective Persulfide Detection Reveals Evolutionarily Conserved Antiaging Effects of S-Sulfhydration. Cell Metab..

[26] Sahu A.K., Minetti A., Di Fraia D., Marino A., Winterhalter P.R., Giustarini D., Rossi R., Simm A., Neri F., Galvagni F. (2026). Oxidative stress causes a reversible decrease of deubiquitylases activity in old vertebrate brains. Nat. Commun..

[27] Labbadia J., Morimoto R.I. (2015). The biology of proteostasis in aging and disease. Annu. Rev. Biochem..

[28] Jayaraj G.G., Hipp M.S., Hartl F.U. (2020). Functional Modules of the Proteostasis Network. Cold Spring Harb. Perspect. Biol..

[29] Benchoam D., Cuevasanta E., Roman J.V., Banerjee R., Alvarez B. (2024). Acidity of persulfides and its modulation by the protein environments in sulfide quinone oxidoreductase and thiosulfate sulfurtransferase. J. Biol. Chem..

[30] Cuevasanta E., Lange M., Bonanata J., Coitino E.L., Ferrer-Sueta G., Filipovic M.R., Alvarez B. (2015). Reaction of Hydrogen Sulfide with Disulfide and Sulfenic Acid to Form the Strongly Nucleophilic Persulfide. J. Biol. Chem..

[31] Wedmann R., Onderka C., Wei S., Szijarto I.A., Miljkovic J.L., Mitrovic A., Lange M., Savitsky S., Yadav P.K., Torregrossa R. (2016). Improved tag-switch method reveals that thioredoxin acts as depersulfidase and controls the intracellular levels of protein persulfidation. Chem. Sci..

[32] Doka E., Pader I., Biro A., Johansson K., Cheng Q., Ballago K., Prigge J.R., Pastor-Flores D., Dick T.P., Schmidt E.E. (2016). A novel persulfide detection method reveals protein persulfide- and polysulfide-reducing functions of thioredoxin and glutathione systems. Sci. Adv..

[33] Zhang D., Macinkovic I., Devarie-Baez N.O., Pan J., Park C.M., Carroll K.S., Filipovic M.R., Xian M. (2014). Detection of protein S-sulfhydration by a tag-switch technique. Angew. Chem. Int. Ed..

[34] Pan J., Carroll K.S. (2013). Persulfide reactivity in the detection of protein s-sulfhydration. ACS Chem. Biol..

[35] Soito L., Williamson C., Knutson S.T., Fetrow J.S., Poole L.B., Nelson K.J. (2011). PREX: PeroxiRedoxin classification indEX, a database of subfamily assignments across the diverse peroxiredoxin family. Nucleic Acids Res..

[36] Woo H.A., Jeong W., Chang T.S., Park K.J., Park S.J., Yang J.S., Rhee S.G. (2005). Reduction of cysteine sulfinic acid by sulfiredoxin is specific to 2-cys peroxiredoxins. J. Biol. Chem..

[37] Biteau B., Labarre J., Toledano M.B. (2003). ATP-dependent reduction of cysteine-sulphinic acid by *S. cerevisiae* sulphiredoxin. Nature.

[38] Martinez-Ruiz A., Villanueva L., Gonzalez de Orduna C., Lopez-Ferrer D., Higueras M.A., Tarin C., Rodriguez-Crespo I., Vazquez J., Lamas S. (2005). S-nitrosylation of Hsp90 promotes the inhibition of its ATPase and endothelial nitric oxide synthase regulatory activities. Proc. Natl. Acad. Sci. USA.

[39] Ahn S.G., Thiele D.J. (2003). Redox regulation of mammalian heat shock factor 1 is essential for Hsp gene activation and protection from stress. Genes. Dev..

[40] Hugo M., Korovila I., Kohler M., Garcia-Garcia C., Cabrera-Garcia J.D., Marina A., Martinez-Ruiz A., Grune T. (2018). Early cysteine-dependent inactivation of 26S proteasomes does not involve particle disassembly. Redox Biol..

[41] Livnat-Levanon N., Kevei E., Kleifeld O., Krutauz D., Segref A., Rinaldi T., Erpapazoglou Z., Cohen M., Reis N., Hoppe T. (2014). Reversible 26S proteasome disassembly upon mitochondrial stress. Cell Rep..

[42] Kelmer Sacramento E., Kirkpatrick J.M., Mazzetto M., Baumgart M., Bartolome A., Di Sanzo S., Caterino C., Sanguanini M., Papaevgeniou N., Lefaki M. (2020). Reduced proteasome activity in the aging brain results in ribosome stoichiometry loss and aggregation. Mol. Syst. Biol..

[43] Alberti S., Hyman A.A. (2021). Biomolecular condensates at the nexus of cellular stress, protein aggregation disease and ageing. Nat. Rev. Mol. Cell Biol..

[44] Boeynaems S., Alberti S., Fawzi N.L., Mittag T., Polymenidou M., Rousseau F., Schymkowitz J., Shorter J., Wolozin B., Van Den Bosch L. (2018). Protein Phase Separation: A New Phase in Cell Biology. Trends Cell Biol..

[45] Banani S.F., Lee H.O., Hyman A.A., Rosen M.K. (2017). Biomolecular condensates: Organizers of cellular biochemistry. Nat. Rev. Mol. Cell Biol..

[46] Alberti S., Gladfelter A., Mittag T. (2019). Considerations and Challenges in Studying Liquid-Liquid Phase Separation and Biomolecular Condensates. Cell.

[47] Zbinden A., Perez-Berlanga M., De Rossi P., Polymenidou M. (2020). Phase Separation and Neurodegenerative Diseases: A Disturbance in the Force. Dev. Cell.

[48] Ambadipudi S., Biernat J., Riedel D., Mandelkow E., Zweckstetter M. (2017). Liquid-liquid phase separation of the microtubule-binding repeats of the Alzheimer-related protein Tau. Nat. Commun..

[49] Ghosh U., Tse E., Yang H., Shi M., Carlo C.D., Wang F., Merz G.E., Prusiner S.B., Southworth D.R., Condello C. (2024). Cryo-EM Structures Reveal Tau Filaments from Down Syndrome Adopt Alzheimer’s Disease Fold. bioRxiv.

[50] Nizynski B., Dzwolak W., Nieznanski K. (2017). Amyloidogenesis of Tau protein. Protein Sci..

[51] Orr M.E., Sullivan A.C., Frost B. (2017). A Brief Overview of Tauopathy: Causes, Consequences, and Therapeutic Strategies. Trends Pharmacol. Sci..

[52] Hernandez-Vega A., Braun M., Scharrel L., Jahnel M., Wegmann S., Hyman B.T., Alberti S., Diez S., Hyman A.A. (2017). Local Nucleation of Microtubule Bundles through Tubulin Concentration into a Condensed Tau Phase. Cell Rep..

[53] Zhang X., Lin Y., Eschmann N.A., Zhou H., Rauch J.N., Hernandez I., Guzman E., Kosik K.S., Han S. (2017). RNA stores tau reversibly in complex coacervates. PLoS Biol..

[54] Piroska L., Fenyi A., Thomas S., Plamont M.A., Redeker V., Melki R., Gueroui Z. (2023). alpha-Synuclein liquid condensates fuel fibrillar alpha-synuclein growth. Sci. Adv..

[55] Ray S., Singh N., Kumar R., Patel K., Pandey S., Datta D., Mahato J., Panigrahi R., Navalkar A., Mehra S. (2020). alpha-Synuclein aggregation nucleates through liquid-liquid phase separation. Nat. Chem..

[56] Aktar F., Burudpakdee C., Polanco M., Pei S., Swayne T.C., Lipke P.N., Emtage L. (2019). The huntingtin inclusion is a dynamic phase-separated compartment. Life Sci. Alliance.

[57] Peskett T.R., Rau F., O’Driscoll J., Patani R., Lowe A.R., Saibil H.R. (2018). A Liquid to Solid Phase Transition Underlying Pathological Huntingtin Exon1 Aggregation. Mol. Cell.

[58] Posey A.E., Ruff K.M., Harmon T.S., Crick S.L., Li A., Diamond M.I., Pappu R.V. (2018). Profilin reduces aggregation and phase separation of huntingtin N-terminal fragments by preferentially binding to soluble monomers and oligomers. J. Biol. Chem..

[59] Afroz T., Hock E.M., Ernst P., Foglieni C., Jambeau M., Gilhespy L.A.B., Laferriere F., Maniecka Z., Pluckthun A., Mittl P. (2017). Functional and dynamic polymerization of the ALS-linked protein TDP-43 antagonizes its pathologic aggregation. Nat. Commun..

[60] Carey J.L., Guo L. (2022). Liquid-Liquid Phase Separation of TDP-43 and FUS in Physiology and Pathology of Neurodegenerative Diseases. Front. Mol. Biosci..

[61] Conicella A.E., Dignon G.L., Zerze G.H., Schmidt H.B., D’Ordine A.M., Kim Y.C., Rohatgi R., Ayala Y.M., Mittal J., Fawzi N.L. (2020). TDP-43 alpha-helical structure tunes liquid-liquid phase separation and function. Proc. Natl. Acad. Sci. USA.

[62] Conicella A.E., Zerze G.H., Mittal J., Fawzi N.L. (2016). ALS Mutations Disrupt Phase Separation Mediated by alpha-Helical Structure in the TDP-43 Low-Complexity C-Terminal Domain. Structure.

[63] Dormann D., Haass C. (2011). TDP-43 and FUS: A nuclear affair. Trends Neurosci..

[64] Gopal P.P., Nirschl J.J., Klinman E., Holzbaur E.L. (2017). Amyotrophic lateral sclerosis-linked mutations increase the viscosity of liquid-like TDP-43 RNP granules in neurons. Proc. Natl. Acad. Sci. USA.

[65] Li H.R., Chiang W.C., Chou P.C., Wang W.J., Huang J.R. (2018). TAR DNA-binding protein 43 (TDP-43) liquid-liquid phase separation is mediated by just a few aromatic residues. J. Biol. Chem..

[66] Neumann M., Sampathu D.M., Kwong L.K., Truax A.C., Micsenyi M.C., Chou T.T., Bruce J., Schuck T., Grossman M., Clark C.M. (2006). Ubiquitinated TDP-43 in frontotemporal lobar degeneration and amyotrophic lateral sclerosis. Science.

[67] Wang A., Conicella A.E., Schmidt H.B., Martin E.W., Rhoads S.N., Reeb A.N., Nourse A., Ramirez Montero D., Ryan V.H., Rohatgi R. (2018). A single N-terminal phosphomimic disrupts TDP-43 polymerization, phase separation, and RNA splicing. EMBO J..

[68] Patel A., Lee H.O., Jawerth L., Maharana S., Jahnel M., Hein M.Y., Stoynov S., Mahamid J., Saha S., Franzmann T.M. (2015). A Liquid-to-Solid Phase Transition of the ALS Protein FUS Accelerated by Disease Mutation. Cell.

[69] Batlle C., Yang P., Coughlin M., Messing J., Pesarrodona M., Szulc E., Salvatella X., Kim H.J., Taylor J.P., Ventura S. (2020). hnRNPDL Phase Separation Is Regulated by Alternative Splicing and Disease-Causing Mutations Accelerate Its Aggregation. Cell Rep..

[70] Garcia-Pardo J., Bartolome-Nafria A., Chaves-Sanjuan A., Gil-Garcia M., Visentin C., Bolognesi M., Ricagno S., Ventura S. (2023). Cryo-EM structure of hnRNPDL-2 fibrils, a functional amyloid associated with limb-girdle muscular dystrophy D3. Nat. Commun..

[71] Garcia-Pardo J., Ventura S. (2023). Cryo-EM structures of functional and pathological amyloid ribonucleoprotein assemblies. Trends Biochem. Sci..

[72] Bartolome-Nafria A., Garcia-Pardo J., Ventura S. (2024). Mutations in human prion-like domains: Pathogenic but not always amyloidogenic. Prion.

[73] Borkosky S.S., Peralta-Martinez R., Armella-Sierra A., Esperante S.A., Lizarraga L., Garcia-Pardo J., Ventura S., Sanchez I.E., de Prat-Gay G. (2025). Experimental kinetic mechanism of P53 condensation-amyloid aggregation. Biophys. J..

[74] Datta D., Navalkar A., Sakunthala A., Paul A., Patel K., Masurkar S., Gadhe L., Manna S., Bhattacharyya A., Sengupta S. (2024). Nucleo-cytoplasmic environment modulates spatiotemporal p53 phase separation. Sci. Adv..

[75] Petronilho E.C., de Andrade G.C., de Sousa G.D.S., Almeida F.P., Mota M.F., Gomes A., Pinheiro C.H.S., da Silva M.C., Arruda H.R.S., Marques M.A. (2024). Oncogenic p53 triggers amyloid aggregation of p63 and p73 liquid droplets. Commun. Chem..

[76] Petronilho E.C., Pedrote M.M., Marques M.A., Passos Y.M., Mota M.F., Jakobus B., de Sousa G.D.S., Pereira da Costa F., Felix A.L., Ferretti G.D.S. (2021). Phase separation of p53 precedes aggregation and is affected by oncogenic mutations and ligands. Chem. Sci..

[77] Marino A., Di Fraia D., Panfilova D., Sahu A.K., Minetti A., Omrani O., Cirri E., Ori A. (2025). Aging and diet alter the protein ubiquitylation landscape in the mouse brain. Nat. Commun..

[78] Koyuncu S., Loureiro R., Lee H.J., Wagle P., Krueger M., Vilchez D. (2021). Rewiring of the ubiquitinated proteome determines ageing in *C. elegans*. Nature.

[79] Basisty N., Meyer J.G., Schilling B. (2018). Protein Turnover in Aging and Longevity. Proteomics.

[80] Mishra M., Wu J., Kane A.E., Howlett S.E. (2024). The intersection of frailty and metabolism. Cell Metab..

[81] Shen X., Wang C., Zhou X., Zhou W., Hornburg D., Wu S., Snyder M.P. (2024). Nonlinear dynamics of multi-omics profiles during human aging. Nat. Aging.

[82] Chakraborty S., Jamuna Tripathi S., Vazquez-Rosa E., Chaubey K., Fujioka H., Miller E., Tyagi R., Vignane T., Sharma S.M., Thomas B. (2025). Cystathionine gamma-lyase is a major regulator of cognitive function through neurotrophin signaling and neurogenesis. Proc. Natl. Acad. Sci. USA.

[83] Jung T., Bader N., Grune T. (2007). Lipofuscin: Formation, distribution, and metabolic consequences. Ann. N. Y. Acad. Sci..

[84] Konig J., Ott C., Hugo M., Jung T., Bulteau A.L., Grune T., Hohn A. (2017). Mitochondrial contribution to lipofuscin formation. Redox Biol..

[85] Giovinazzo D., Bursac B., Sbodio J.I., Nalluru S., Vignane T., Snowman A.M., Albacarys L.M., Sedlak T.W., Torregrossa R., Whiteman M. (2021). Hydrogen sulfide is neuroprotective in Alzheimer’s disease by sulfhydrating GSK3beta and inhibiting Tau hyperphosphorylation. Proc. Natl. Acad. Sci. USA.

[86] Paul B.D., Sbodio J.I., Xu R., Vandiver M.S., Cha J.Y., Snowman A.M., Snyder S.H. (2014). Cystathionine gamma-lyase deficiency mediates neurodegeneration in Huntington’s disease. Nature.

[87] Snijder P.M., Baratashvili M., Grzeschik N.A., Leuvenink H.G.D., Kuijpers L., Huitema S., Schaap O., Giepmans B.N.G., Kuipers J., Miljkovic J.L. (2016). Overexpression of Cystathionine gamma-Lyase Suppresses Detrimental Effects of Spinocerebellar Ataxia Type 3. Mol. Med..

[88] Cirino G., Szabo C., Papapetropoulos A. (2023). Physiological roles of hydrogen sulfide in mammalian cells, tissues, and organs. Physiol. Rev..

[89] Sen N., Paul B.D., Gadalla M.M., Mustafa A.K., Sen T., Xu R., Kim S., Snyder S.H. (2012). Hydrogen sulfide-linked sulfhydration of NF-kappaB mediates its antiapoptotic actions. Mol. Cell.

[90] Yang G., Zhao K., Ju Y., Mani S., Cao Q., Puukila S., Khaper N., Wu L., Wang R. (2013). Hydrogen sulfide protects against cellular senescence via S-sulfhydration of Keap1 and activation of Nrf2. Antioxid. Redox Signal..

[91] Petrovic D., Slade L., Paikopoulos Y., D’Andrea D., Savic N., Stancic A., Miljkovic J.L., Vignane T., Drekolia M.K., Mladenovic D. (2025). Ergothioneine improves healthspan of aged animals by enhancing cGPDH activity through CSE-dependent persulfidation. Cell Metab..

[92] Li L., Whiteman M., Guan Y.Y., Neo K.L., Cheng Y., Lee S.W., Zhao Y., Baskar R., Tan C.H., Moore P.K. (2008). Characterization of a novel, water-soluble hydrogen sulfide-releasing molecule (GYY4137): New insights into the biology of hydrogen sulfide. Circulation.

[93] Gero D., Torregrossa R., Perry A., Waters A., Le-Trionnaire S., Whatmore J.L., Wood M., Whiteman M. (2016). The novel mitochondria-targeted hydrogen sulfide (H_2_S) donors AP123 and AP39 protect against hyperglycemic injury in microvascular endothelial cells in vitro. Pharmacol. Res..

[94] Le Trionnaire S., Perry A., Szczesny B., Szabo C., Winyard P.G., Whatmore J.L., Wood M.E., Whiteman M. (2014). The synthesis and functional evaluation of a mitochondria-targeted hydrogen sulfide donor, (10-oxo-10-(4-(3-thioxo-3-1,2-dithiol-5-yl)phenoxy)decyl)triphenylphosphonium bromide (AP39). Medchemcomm.

[95] Polhemus D.J., Li Z., Pattillo C.B., Gojon G., Gojon G., Giordano T., Krum H. (2015). A novel hydrogen sulfide prodrug, SG1002, promotes hydrogen sulfide and nitric oxide bioavailability in heart failure patients. Cardiovasc. Ther..

[96] Benavides G.A., Squadrito G.L., Mills R.W., Patel H.D., Isbell T.S., Patel R.P., Darley-Usmar V.M., Doeller J.E., Kraus D.W. (2007). Hydrogen sulfide mediates the vasoactivity of garlic. Proc. Natl. Acad. Sci. USA.

[97] Hine C., Harputlugil E., Zhang Y., Ruckenstuhl C., Lee B.C., Brace L., Longchamp A., Trevino-Villarreal J.H., Mejia P., Ozaki C.K. (2015). Endogenous hydrogen sulfide production is essential for dietary restriction benefits. Cell.

[98] Ghaffari Zaki A., Issa H., Miri S.M., Armouch J., Aydeger A., Yildirim S., Mete R.Z., Aljundi O., Vatandaslar E., Akgul Caglar T. (2026). Chemogenetic Manipulation of H_2_S with Spatiotemporal Precision. Precis. Chem..

[99] Kabil H., Kabil O., Banerjee R., Harshman L.G., Pletcher S.D. (2011). Increased transsulfuration mediates longevity and dietary restriction in Drosophila. Proc. Natl. Acad. Sci. USA.

[100] Yang G., Wu L., Jiang B., Yang W., Qi J., Cao K., Meng Q., Mustafa A.K., Mu W., Zhang S. (2008). H_2_S as a physiologic vasorelaxant: Hypertension in mice with deletion of cystathionine gamma-lyase. Science.

[101] Roorda M., Miljkovic J.L., van Goor H., Henning R.H., Bouma H.R. (2021). Spatiotemporal regulation of hydrogen sulfide signaling in the kidney. Redox Biol..

[102] Modis K., Bos E.M., Calzia E., van Goor H., Coletta C., Papapetropoulos A., Hellmich M.R., Radermacher P., Bouillaud F., Szabo C. (2014). Regulation of mitochondrial bioenergetic function by hydrogen sulfide. Part II. Pathophysiological and therapeutic aspects. Br. J. Pharmacol..

[103] Panagaki T., Randi E.B., Augsburger F., Szabo C. (2019). Overproduction of H_2_S, generated by CBS, inhibits mitochondrial Complex IV and suppresses oxidative phosphorylation in Down syndrome. Proc. Natl. Acad. Sci. USA.

[104] Forman H.J., Zhang H. (2021). Targeting oxidative stress in disease: Promise and limitations of antioxidant therapy. Nat. Rev. Drug Discov..

[105] Bourgonje A.R., Mann G.E., van Goor H. (2026). Redox medicine: Translation of basic science to the clinic. Free Radic. Biol. Med..

[106] Grundemann D., Hartmann L., Flogel S. (2022). The ergothioneine transporter (ETT): Substrates and locations, an inventory. FEBS Lett..

[107] Halliwell B., Tang R.M.Y., Cheah I.K. (2023). Diet-Derived Antioxidants: The Special Case of Ergothioneine. Annu. Rev. Food Sci. Technol..

[108] Paul B.D., Snyder S.H. (2010). The unusual amino acid L-ergothioneine is a physiologic cytoprotectant. Cell Death Differ..

[109] Liu S., Fu S., Wang G., Cao Y., Li L., Li X., Yang J., Li N., Shan Y., Cao Y. (2021). Glycerol-3-phosphate biosynthesis regenerates cytosolic NAD^+^ to alleviate mitochondrial disease. Cell Metab..

[110] Covarrubias A.J., Perrone R., Grozio A., Verdin E. (2021). NAD^+^ metabolism and its roles in cellular processes during ageing. Nat. Rev. Mol. Cell Biol..

[111] Cheah I.K., Halliwell B. (2021). Ergothioneine, recent developments. Redox Biol..

[112] Halliwell B., Cheah I. (2024). Are age-related neurodegenerative diseases caused by a lack of the diet-derived compound ergothioneine?. Free Radic. Biol. Med..

[113] Reeg S., Castro J.P., Hugo M., Grune T. (2020). Accumulation of polyubiquitinated proteins: A consequence of early inactivation of the 26S proteasome. Free Radic. Biol. Med..

